# Genetic variants associated with glaucomatous visual field loss in primary open-angle glaucoma

**DOI:** 10.1038/s41598-022-24915-x

**Published:** 2022-12-01

**Authors:** Fumihiko Mabuchi, Nakako Mabuchi, Yoichi Sakurada, Seigo Yoneyama, Kenji Kashiwagi, Zentaro Yamagata, Mitsuko Takamoto, Makoto Aihara, Takeshi Iwata, Kazuki Hashimoto, Kota Sato, Yukihiro Shiga, Toru Nakazawa, Masato Akiyama, Kazuhide Kawase, Mineo Ozaki, Makoto Araie

**Affiliations:** 1 Department of Ophthalmology, Faculty of Medicine, University of Yamanashi, Chuo, Yamanashi, Japan; 2 Department of Health Sciences, Faculty of Medicine, University of Yamanashi, Chuo, Yamanashi, Japan; 3 Department of Ophthalmology, Saitama Red Cross Hospital, Chuo-ku, Saitama, Japan; 4 Department of Ophthalmology, Graduate School of Medicine, University of Tokyo, Bunkyo-ku, Tokyo, Japan; 5 Division of Molecular and Cellular Biology, National Institute of Sensory Organs, National Hospital Organization Tokyo Medical Center, Meguro-ku, Tokyo, Japan; 6 Department of Ophthalmology, Tohoku University Graduate School of Medicine, Sendai, Miyagi Japan; 7 Department of Ophthalmic Imaging and Information Analytics, Tohoku University Graduate School of Medicine, Sendai, Miyagi Japan; 8 Collaborative Program for Ophthalmic Drug Discovery, Tohoku University Graduate School of Medicine, Sendai, Miyagi Japan; 9 Department of Ocular Pathology and Imaging Science, Graduate School of Medical Sciences, Kyushu University, Fukuoka City, Fukuoka, Japan; 10 Yasuma Eye Clinic, Nagoya, Aichi Japan; 11 Department of Ophthalmology Protective Care for Sensory Disorders, Nagoya University Graduate School of Medicine, Nagoya, Aichi Japan; 12 Ozaki Eye Hospital, Hyuga, Miyazaki Japan; 13 Kanto Central Hospital of the Mutual Aid Association of Public School Teachers, Setagaya-ku, Tokyo, Japan

**Keywords:** Glaucoma, Disease genetics

## Abstract

Primary open-angle glaucoma (POAG) is characterized by a progressive optic neuropathy with visual field loss. To investigate the genetic variants associated with visual field loss in POAG, Japanese POAG patients (n = 426) and control subjects (n = 246) were genotyped for 22 genetic variants predisposing to POAG that can be classified into those associated with intraocular pressure (IOP) elevation (IOP-related genetic variants) and optic nerve vulnerability independent of IOP (optic nerve-related genetic variants). The genetic risk score (GRS) of the 17 IOP-related and five optic nerve-related genetic variants was calculated, and the associations between the GRS and the mean deviation (MD) of automated static perimetry as an indicator of the severity of visual field loss and pattern standard deviation (PSD) as an indicator of the focal disturbance were evaluated. There was a significant association (Beta = − 0.51, P = 0.0012) between the IOP-related GRS and MD. The severity of visual field loss may depend on the magnitude of IOP elevation induced by additive effects of IOP-related genetic variants. A significant association (n = 135, Beta = 0.65, P = 0.0097) was found between the optic nerve-related, but not IOP-related, GRS and PSD. The optic nerve-related (optic nerve vulnerability) and IOP-related (IOP elevation) genetic variants may play an important role in the focal and diffuse visual field loss respectively. To our knowledge, this is the first report to show an association between additive effects of genetic variants predisposing to POAG and glaucomatous visual field loss, including severity and focal/diffuse disturbance of visual field loss, in POAG.

## Introduction

Glaucoma is characterized by a chronic progressive optic neuropathy with corresponding and characteristic patterns of visual field loss. In most cases, glaucomatous visual field loss is initially localized in the nasal or in the arcuate region and as the disease progresses, the focal loss becomes wider, deeper, and more numerous. Finally, some cases become blind even while they are receiving therapy.

Primary open-angle glaucoma (POAG) represents the most prevalent form of glaucoma, and clinically, intraocular pressure (IOP) elevation and myopia are reported to be risk factors for optic nerve damage in POAG^1^. Additionally, a positive family history of glaucoma is a major risk factor for POAG^1–6^, and genetic factors are therefore considered to play an important role in the pathogenesis of POAG. Genetic analyses, including genome-wide association study (GWAS), have recently identified genetic variants predisposing to POAG^7–28^. These genetic variants can be classified into two types: one type involves genetic variants associated with IOP elevation (IOP-related genetic variants); the other involves genetic variants associated with vulnerability of the optic nerve, independent of IOP (optic nerve-related genetic variants), which may include genetic variants associated with apoptosis of optic nerve^29^, myopia^1^, and optic nerve circulation^30^. Moreover, previous studies have reported the additive effects of these genetic variants on clinical features, such as phenotypes including normal tension glaucoma (NTG) and high tension glaucoma (HTG)^19,26,27,31–34^, vertical cup-to-disc ratio (VCDR)^35^, IOP^23,36,37^, family history of glaucoma^27,33,37,38^, age at diagnosis of glaucoma^27,37–39^, number of medications^37^, and surgical intervention^27,37^. However, an association between the additive effects of genetic variants predisposing to POAG and glaucomatous visual field loss, the most important clinical symptom in POAG, has not been found.

In order to further elucidate the genetic mechanism of visual field loss in POAG, the present study was conducted to investigate the association between the IOP-related/optic nerve-related genetic variants and the mean deviation (MD) of automated static perimetry as an indicator of the severity of visual field loss and the pattern standard deviation (PSD) as an indicator of the focal visual field loss.

## Results

Six hundred seventy-two Japanese patients, including 426 patients with POAG (HTG, n = 210; NTG, n = 216) and 246 control subjects, were enrolled in the present study. The demographic and clinical data for all participants are shown in Table 1. The mean age at the blood sampling was 63.1 ± 13.6 years (standard deviation) in patients with POAG and 67.7 ± 11.2 years in the control subjects. The mean of maximum IOP was 23.4 ± 7.7 mmHg in patients with POAG and 15.0 ± 2.6 mmHg in the control subjects.

**Table 1:** Demographic and clinical data in patients with primary open-angle glaucoma and control subjects.

Clinical values	Control (n = 246)	POAG (n = 426)	P value	Early to moderate stage POAG (n = 135)	P value
Age at blood sampling, years	67.7 ± 11.2	63.1 ± 13.6	< 0.001	58.8 ± 13.1	< 0.001
Age at diagnosis of glaucoma, years	–	56.1 ± 13.9	–	52.9 ± 12.7	–
Men, n (%)	90 (36.6)	210 (49.3)	0.0017	60 (44.4)	0.15
Refractive error, diopter	− 0.2 ± 2.0	− 2.3 ± 3.4	< 0.001	− 2.3 ± 3.2	< 0.001
Maximum IOP, mmHg	15.0 ± 2.6	23.4 ± 7.7	< 0.001	21.3 ± 4.9	< 0.001
MD of HFA30-2* in the worse eye, dB	–	− 15.1 ± 8.0	–	− 6.9 ± 2.6	–
PSD of HFA30-2* in the worse eye, dB	–	− 10.4 ± 3.4	–	− 9.1 ± 3.2	–
NTG, n (%)	–	216 (50.7)	–	85 (63.0)	–
Positive family history of glaucoma, n (%)	0 (0)	113 (26.5)	–	41 (30.4)	–

Early to moderate stage POAG is a subset used for PSD analyses. Continuous variables are expressed as mean ± standard deviation. Fisher exact test for comparison of proportion and Student t test for continuous variables.

*POAG* primary open-angle glaucoma, *IOP* intraocular pressure, *MD* mean deviation, *HFA30-2* Humphrey field analyzer 30–2, *PSD* pattern standard deviation, *NTG* normal tension glaucoma.

*Automated static perimetry.

### Association between the genetic risk score (GRS) and MD

**Figure 1:**
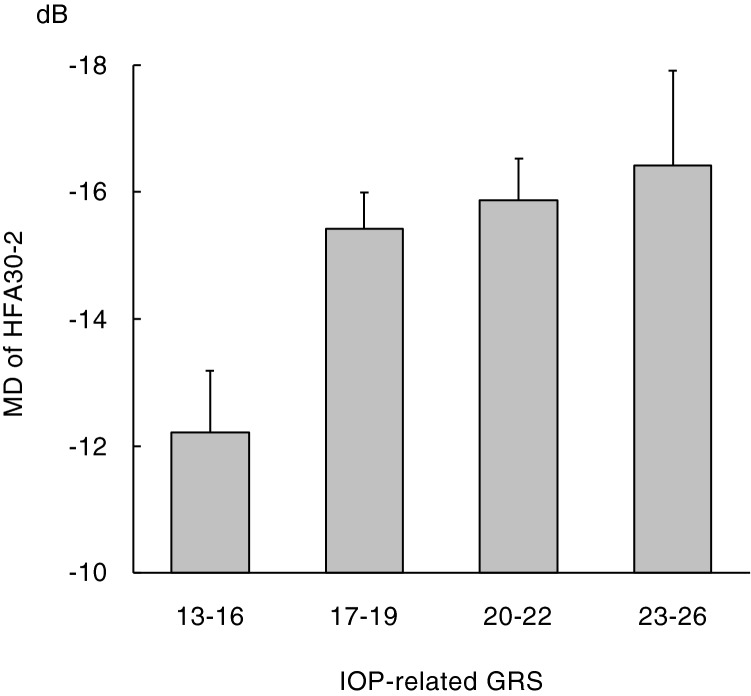
Mean MD values of automated static perimetry (HFA30-2) in the worse eye divided by the GRS of IOP-related genetic variants in patients with primary open-angle glaucoma. As the GRS of IOP-related genetic variants increased, the mean MD values decreased. *MD* mean deviation, *HFA30-2* Humphrey field analyzer 30–2, *IOP* intraocular pressure, *GRS* genetic risk score.

The mean MD of automated static perimetry (Humphrey Field Analyzer 30–2: HFA30-2, Humphrey Instruments, San Leandro, CA) in the worse eye were -15.1 ± 8.0 dB in patients with POAG. The results of a multiple linear regression analysis with the MD as a dependent variable and age, sex and the GRS as independent variables are shown in Table 2. There was a significant association (Beta = − 0.51, 95% confidence interval CI  − 0.81 to − 0.20, P = 0.0012) between the GRS of IOP-related genetic variants and the MD. As the GRS of IOP-related genetic variants increased, the MD decreased. A graphical representation of mean MD values divided by the GRS of IOP-related genetic variants is shown in Fig. 1.

**Table 2:** Results of a multiple linear regression analysis with mean deviation of automated static perimetry* in the worse eye as a dependent variable in patients with primary open-angle glaucoma.

Independent variables	Beta^†^ (95% CI)	SE	P value
Age, years	− 0.17 to − 0.16 (− 0.22 to − 0.11)	0.027–0.028	< 0.001
Male sex	− 2.07 to − 1.93 (− 3.55 to − 0.47)	0.74–0.75	0.0059–0.0095
GRS of 5 optic nerve-related genetic variants	0.047 (− 0.60 to 0.69)	0.33	0.89
GRS of 17 IOP-related genetic variants	− 0.51 (− 0.81 to − 0.20)	0.15	0.0012

*CI* confidence interval, *SE* standard error, *GRS* genetic risk score, *IOP* intraocular pressure.

*Humphrey field analyzer 30–2 (HFA30-2), ^†^Regression coefficient.

F change = 13.1–17.0, P < 0.001.

### Association between the GRS and PSD

**Figure 2:**
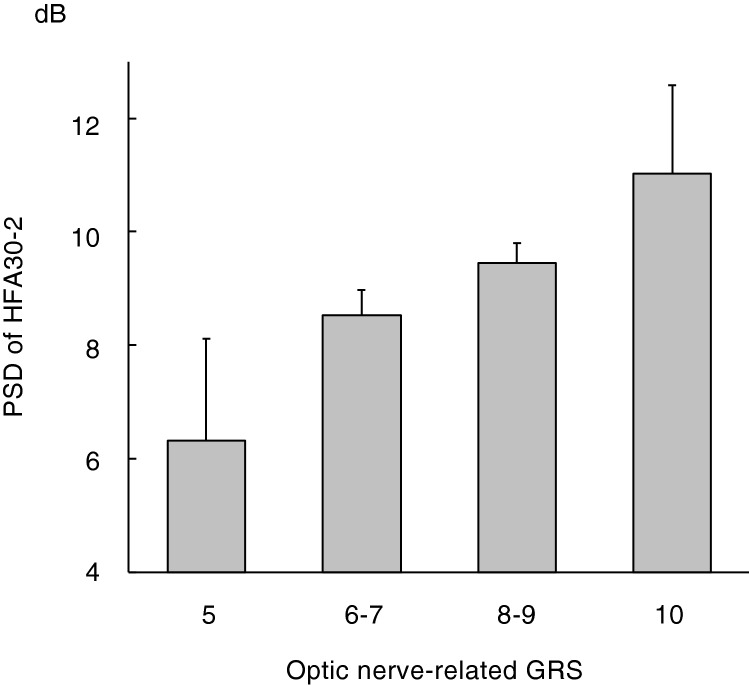
Mean PSD values of automated static perimetry (HFA30-2) in the worse eye divided by the GRS of optic nerve-related genetic variants in patients with primary open-angle glaucoma. As the GRS of optic nerve-related genetic variants increased, the mean PSD values increased. *PSD* patten standard deviation, *HFA30-2* Humphrey field analyzer 30–2, *GRS* genetic risk score.

One hundred thirty-five patients with early to moderate stage POAG were enrolled in this analysis. The mean MD and PSD of HFA30-2 in the worse eye were − 6.9 ± 2.6 and 9.1 ± 3.2 dB respectively. The results of a multiple linear regression analysis with the PSD as a dependent variable and age, sex and the GRS as independent variables are shown in Table 3. There was a significant association (Beta = 0.65, 95% CI 0.16–1.15, P = 0.0097) between the GRS of optic nerve-related genetic variants and the PSD. As the GRS of optic nerve-related genetic variants increased, the PSD increased. A graphical representation of mean PSD values divided by the GRS of optic nerve-related genetic variants is shown in Fig. 2.

**Table 3:** Results of a multiple linear regression analysis with pattern standard deviation of automated static perimetry* in the worse eye as a dependent variable in patients with primary open-angle glaucoma.

Independent variables	Beta^†^ (95% CI)	SE	P value
Age, years	− 0.026 to − 0.014 (− 0.056 to 0.029)	0.021	0.23–0.52
Male sex	− 1.04 to − 0.74 (− 2.16 to 0.37)	0.56–0.57	0.068–0.19
GRS of 5 optic nerve-related genetic variants	0.65 (0.16–1.15)	0.25	0.0097
GRS of 17 IOP-related genetic variants	0.15 (− 0.064 to 0.36)	0.11	0.17

*CI* confidence interval, *SE* standard error, *GRS* genetic risk score, *IOP* intraocular pressure.

*Humphrey field analyzer 30–2 (HFA30-2), ^†^Regression coefficient.

F change = 1.3–2.9, P = 0.035–0.29.

## Discussion

In the present study, we investigated the association between the IOP-related/optic nerve-related genetic variants and MD as an indicator of the severity or PSD as an indicator of the focal disturbance of glaucomatous visual field loss in POAG. There was a significant association between the IOP-related GRS and MD, and as the IOP-related GRS increased, the MD decreased. This result indicates that the severity (MD) of glaucomatous visual field loss may depend on the magnitude of IOP elevation induced by additive effects of IOP-related genetic variants. It may be reasonable, as it is clinically reported that reducing the IOP in glaucomatous eyes prevents disease progression. The IOP-related GRS is also reported to be associated with age at the diagnosis of glaucoma as an indicator of the progression of POAG^27,38^, which may support the results of the present study. No significant association has previously been found between the additive effects of IOP-related genetic variants and MD^37^. The POAG patients with a wider range of maximum IOP (IOP-related GRS) might be included in the present study because the prevalence of NTG in the Japanese population is higher than that in other ethnic populations^40^. This may be the reason why a significant association could be found between them in the present study. Previous studies have reported the association between the genetic variants near *CAV1/CAV2*^41^ or *p53*^42^ and POAG with paracentral visual field loss. In the present study, a significant association was found between the optic nerve-related GRS and PSD. As the optic nerve-related GRS increased, the PSD increased. This result indicates that the additive effects of optic nerve-related genetic variants are associated with focal glaucomatous visual field loss. It has been reported that focal glaucomatous visual field loss occurs at a lower IOP than diffuse loss and—as such—may be a marker that can be used to identify patients whose optic nerves are abnormally susceptible to glaucomatous injury^43^. Focal glaucomatous visual field loss may occur due to vulnerability of the optic nerve induced by additive effects of optic nerve-related genetic variants. In other words, the optic nerve vulnerability induced by optic nerve-related genetic variants may result in typical glaucomatous visual field loss, such as nasal step and/or partial arcuate visual field loss. In contrast, as described above, the IOP-related GRS was associated with the MD, but not the PSD, which gives an overall value of the total amount of visual function loss, but not the localized visual function loss. A previous study reported that POAG patients with diffuse visual field depression manifested higher IOP than those with localized visual field defects^44^. It was also reported that IOP was significantly higher in patients with generalized enlargement of the optic cup discs, which indicates diffuse glaucomatous visual field loss^45^. These results indicate that IOP-related genetic variants are associated with diffuse glaucomatous visual field loss. On the whole, the optic nerve-related (optic nerve vulnerability) and IOP-related (IOP elevation) genetic variants may contribute to focal and diffuse glaucomatous visual field loss respectively. To our knowledge, this is the first report to show an association between additive effects of genetic variants predisposing to POAG and glaucomatous visual field loss, including severity and focal/diffuse disturbance of visual field loss, in POAG.

To evaluate the additive effects of genetic variants predisposing to POAG, the total number of risk alleles of multi-locus genetic variants was used as an unweighted GRS in the present study. Given that the unweighted GRS approach assumed that all risk alleles had the same magnitude of effect on the risk of POAG, the results might not precisely reflect the additive effects of the genetic variants. Thus, in a previous study that reported the additive effects of genetic variants on the risk of POAG^34^, a logistic regression model was used to estimate the risk (odds ratio) of glaucoma for each risk allele of the genetic variants, and the sum of the logarithmically-converted odds ratios of multi-locus genetic variants was used as a weighted GRS. In the present study, the results obtained using this weighted GRS approach (Supplementary Tables 1, 2) were fundamentally the same as those obtained using the unweighted GRS approach.

With regard to limitations, some genetic variants^16–28,35^ that have been reported to be associated with susceptibility to POAG were not analyzed in the present study. An analysis that includes these genetic variants may be better for evaluating the complex genetic mechanism of POAG, although all reported IOP-related genetic variants should not be included to reduce contamination of optic nerve-related genetic variants in IOP-related genetic variants. GRS studies incorporating additional genetic variants are an important future direction. Media opacity, such as a cataract, has been shown to affect the results of automated static perimetry^46^, and some patients with cataract were included in the present study. To reduce the influence of cataract on the MD and PSD, POAG patients with a best corrected visual acuity of > 20/25 were selected and analyzed. The results obtained using these selected subjects were fundamentally the same as those obtained using the unselected original subjects: the associations between the IOP-related GRS and MD (n = 292, Beta = − 0.36, 95% CI  − 0.67 to − 0.040, P = 0.027), optic nerve-related GRS and PSD (n = 117, Beta = 0.81, 95% CI  0.29–1.33, P = 0.0026). The participants of the present study were all Japanese. Since the genetic background differs between ethnicities, further studies may be necessary to generalize our findings to other ethnic populations.

In summary, glaucomatous visual field loss in cases of POAG is influenced by the genetic variants predisposing to POAG. The severity (MD) of visual field loss was associated with additive effects of IOP-related genetic variants, and therefore accounts for the role of IOP elevation as a risk factor for POAG. The optic nerve-related genetic variants were associated with PSD as an indicator of the focal visual field loss, while the IOP-related genetic variants were not. These results indicate that optic nerve vulnerability to IOP due to optic nerve-related genetic variants may play an important role in the focal visual field loss and that IOP elevation induced by IOP-related genetic variants may play an important role in the diffuse visual field loss in POAG. The present findings are useful for understanding the pathogenesis of glaucomatous visual field loss in POAG.

## Methods

### Subjects

Japanese patients with POAG were recruited from the ophthalmology practices at the Enzan Municipal Hospital, Oizumi Clinic, Uenohara City Hospital, and Yamanashi University Hospital in Yamanashi Prefectures, Japan. POAG was diagnosed when an open anterior chamber angle was detected on a gonioscopic examination, and the typical glaucomatous changes in the optic nerve head (enlargement of the VCDR, and/or focal notching of the optic disc rim, and/or retinal nerve fiber layer defect resulting in a thinning in the neuroretinal rim) with a compatible visual field loss (nasal step and/or partial arcuate visual field loss, etc.) was observed in at least one eye. Anderson-Patella’s criteria^47^ were used to define glaucomatous visual field loss. Briefly, the criteria were as follows: a cluster of ≥ 3 points in the pattern deviation plot in a single hemifield (superior/inferior) with P < 0.05, one of which had to have been P < 0.01, on HFA30-2. In addition, patients were diagnosed with HTG when they had at least one previous IOP measurement of ≥ 22 mmHg with a Goldmann applanation tonometer. Patients with NTG showed an IOP of ≤ 21 mmHg each time they were tested. The highest IOP in both eyes, chosen from all of the measured IOPs in the patient’s medical records was considered to be the maximum IOP, and IOPs measured after surgical treatments were excluded. Patients who had a history of eye surgery, including laser treatment, before the diagnosis of POAG were excluded from the present study. The control subjects, who were recruited from participating institutions to estimate the risk (odds ratio) of glaucoma for each risk allele of genetic variants predisposing to POAG and to calculate a weighted GRS, included Japanese individuals who were over 40 years of age, with an IOP of ≤ 21 mmHg, who exhibited no glaucomatous cupping of the optic disc (no thinning of disc rim and VCDR ≤ 0.4), and who had no family history of glaucoma. Comprehensive ophthalmologic examinations including both slit-lamp biomicroscopy and fundoscopy were performed and written informed consent was obtained from all study participants. The study protocol was prospectively approved by the Ethics Committee of University of Yamanashi, and the present study was conducted in accordance with the Declaration of Helsinki.

### Evaluation of glaucomatous visual field loss

The mean deviation (MD) and pattern standard deviation (PSD) of HFA30-2 in the worse eye were used to evaluate glaucomatous visual field loss in the present study. Eyes with unreliable visual field results defined as > 30% false-negative results, > 30% false-positive results, or > 20% fixation losses were excluded. Eyes with neurological or ocular diseases that could cause visual field loss were also excluded. The number of visual field tests depends on the case, and the latest results within the reliable visual field tests were used for analyses. The MD is an useful indicator that shows a linear change with the progression of glaucoma and was used to evaluate the severity of glaucomatous visual field loss. Blumenthal and associates^48^ reported that the MD value of eyes that are unable to perform automated static perimetry due to poor vision levels corresponds to the value of − 31.43 dB. The MD values of seven eyes that were unable to perform visual field test due to poor vision levels, such as light perception, were assigned values of − 31.43 dB. The PSD is an useful indicator of localized functional loss and was used to evaluate the focal glaucomatous visual field loss. The PSD is based on the pattern deviation plot, and as the visual field loss becomes more diffuse, their values return to normal (toward zero). In fact, the correlations between the MD and PSD values are not linear. Higher PSD values are found with increasing visual field loss, as determined by MD. However, this initial trend is reversed with further functional loss (eyes with MD < − 17 dB approximately)^49^. Thus, PSD is not a good parameter to monitor eyes with advanced POAG. Eyes with early to moderate stage POAG (− 10.99 ≤ MD ≤ − 1.77 dB) were selected to evaluate the association between the PSD and IOP-related/optic nerve-related genetic variants.

### Genomic DNA genotyping

Genomic DNA was purified from peripheral blood with a Flexi Gene® DNA Kit (QIAGEN, Valencia, CA, USA). There are 22 genetic variants that predispose individuals to POAG—17 variants identified as IOP-related genetic variants on GWAS, including rs1052990 (near gene: *CAV2*)^8,50^, rs11656696 (*GAS7*)^51^, rs59072263 (*GLCCI1/ICA1*)^52^, rs2472493 (*ABCA1*)^8–10^, rs58073046 (*ARHGEF12*)^14^, rs2286885 (*FAM125B/LMX1B*)^12,18^, rs8176743 (*ABO*)^8^, rs747782 (*PTPRJ*)^8^, rs4619890 (*AFAP1*)^9^, rs11969985 *(GMDS*)^9^, rs2745572 (*FOXC1*)^15^, rs35934224 (*TXNRD2*)^15^, rs6732795 (*ANTXR1*)^18^, rs9853115 (*DGKG*)^23^, rs10505100 *(ANGPT1*)^23^, rs7924522 (*ETS1*)^23^ and rs61394862 (*ANKH*)^23^ and 5 variants considered to be optic nerve-related genetic variants, including rs3213787 (*SRBD1*)^53^, rs735860 (*ELOVL5*)^53^, rs1063192 (*CDKN2B*)^54^, rs10483727 (*SIX6*)^54^, and rs61861119 (*MYOF*)^23^, were genotyped using TaqMan single nucleotide polymorphism genotyping assays (Applied Biosystems [ABI], Foster City, CA, USA). Assays were performed on a 7300/7500 Real-Time PCR System (ABI, Foster City, CA, USA) according to the manufacturer’s instructions. The frequency of patients with optic nerve-related genetic variants is high in patients with POAG. Similarly, the frequency of patients with high IOP is also high in patients with POAG. The possibility can’t be completely denied that statistically significant association between the optic nerve-related genetic variants and IOP had been found by the confounding effect on GWAS, especially one with higher statistical power by large number of samples, and that optic nerve-related genetic variants had been identified as IOP-related genetic variants. To reduce contamination of optic nerve-related genetic variants in IOP-related genetic variants, the genotyped genetic variants were selected as previously described^38^. Briefly, in addition to the IOP-related genetic variants reported before 2017, the IOP-related genetic variants with top 10 statistically significant association with IOP reported by MacGregor and associates^23^ in 2018 were selected and included in the present study. The IOP-related genetic variants associated with corneal thickness, such as variants near *FNDC3B*^8,55^ and *ADAMTS8*^16,55^, were excluded. The IOP-related genetic variants near *TMCO1*^56^ and *ATXN2*^15^ were not included because these variants were not polymorphic or rare in the Japanese population. The genetic variants near *SRBD1* and *ELOVL5* were included as optic nerve-related variants in the present study because these variants were identified in 2010 on GWAS of Japanese patients with early-onset NTG^53^, in which the IOPs are consistently within the statistically normal range for the general population. The genetic variants near *CDKN2B*, *SIX6*, and *MYOF* were also selected as optic nerve-related variants because these variants were reported to be associated with POAG but not IOP by MacGregor and associates^23^ in 2018 when the present study was conducted.

### Statistical analysis

Data analysis was performed using JMP statistical software version 14.3.0 (SAS Institute Inc., Cary, NC, USA). The demographic and clinical data in patients with POAG and control subjects were compared using Fisher exact test for comparison of proportion and Student t test for continuous variables. To evaluate the additive effects of IOP-related and optic nerve-related genetic variants, the total number of risk alleles of the 17 IOP-related (range: 0–34) and 5 optic nerve-related (range: 0–10) genetic variants were calculated for each participant as a genetic risk score (GRS). To elucidate the genetic variants associated with glaucomatous visual field loss, the associations between the GRS and MD (as an indicator of the severity of visual field loss) or PSD (as an indicator of the focal disturbance of visual field loss) were evaluated using a multiple linear regression analysis adjusted for age and sex. A value of P < 0.05 was considered to be statistically significant.

## Supplementary Information

Supplementary Tables.

## Data Availability

The dataset generated during and/or analyzed during the present study is available in the figshare repository, https://figshare.com/s/74882d717c7a717ee5c6.
