# Supplementary material for: Genetic variants associated with glaucomatous visual field loss in primary open-angle glaucoma
Source: Sci Rep. 2022 Dec 1;12:20744. doi: 10.1038/s41598-022-24915-x (PMC9715669; doi:10.1038/s41598-022-24915-x)
Supplement: Supplementary file 1 — Supplementary Tables. [file 41598_2022_24915_MOESM1_ESM.pdf]

Independent variables	Beta† (95% CI)	SE	P value
Age, years	-0.17 to -0.16 (-0.22 to -0.11)	0.027 to 0.028	P<0.001
Male sex	-2.00 to -1.91 (-3.47 to -0.45)	0.74 to 0.75	0.0080 to 0.011
Weighted$ GRS of 5 optic nerve-related genetic variants	-0.13 (-0.37 to 0.12)	0.12	0.31
Weighted$ GRS of 17 IOP-related genetic variants	-0.19 (-0.32 to -0.054)	0.067	0.0058

Supplementary Table S1. Results of a Multiple Linear Regression Analysis with Mean Deviation of Automated Static Perimetry* in the Worse Eye as a Dependent Variable in Patients with Primary Open-angle Glaucoma. CI: confidence interval, SE: standard error, GRS: genetic risk score, IOP: intraocular pressure. *Humphrey field analyzer 30-2 (HFA30-2), †Regression coefficient, $The risk (odds ratio) of high tension glaucoma for the risk allele of each genetic variant was calculated using a logistic linear regression analysis, and the sum of the logarithmically-converted odds ratios of these genetic variants was used as a weighted GRS. F change=13.5 to 15.9, P<0.001.

Independent variables	Beta† (95% CI)	SE	P value
Age, years	-0.025 to -0.015 (-0.068 to 0.028)	0.021 to 0.027	0.24 to 0.50
Male sex	-1.06 to -0.68 (-2.19 to 0.45)	0.57	0.064 to 0.24
Weighted$ GRS of 5 optic nerve-related genetic variants	0.23 (0.049 to 0.40)	0.089	0.013
Weighted$ GRS of 17 IOP-related genetic variants	0.046 (-0.047 to 0.14)	0.047	0.33

Supplementary Table S2. Results of a Multiple Linear Regression Analysis with Pattern Standard Deviation of Automated Static Perimetry* in the Worse Eye as a Dependent Variable in Patients with Primary Open-angle Glaucoma. CI: confidence interval, SE: standard error, GRS: genetic risk score, IOP: intraocular pressure. *Humphrey field analyzer 30-2 (HFA30-2), †Regression coefficient, $The risk (odds ratio) of high tension glaucoma for the risk allele of each genetic variant was calculated using a logistic linear regression analysis, and the sum of the logarithmically-converted odds ratios of these genetic variants was used as a weighted GRS. F change=0.94 to 2.8, P=0.044 to 0.42.
